# Predictors of overweight/obesity in a Brazilian cohort after 13 years of follow-up

**DOI:** 10.1186/s12937-018-0320-7

**Published:** 2018-01-15

**Authors:** Ludimila Garcia Souza, Thiago Veiga Jardim, Ana Carolina Rezende, Ana Luiza Lima Sousa, Humberto Graner Moreira, Naiana Borges Perillo, Samanta Garcia de Souza, Weimar Kunz Sebba Barroso de Souza, Ymara Cássia Luciana Araújo, Maria do Rosário Gondim Peixoto, Paulo César Brandão Veiga Jardim

**Affiliations:** 1 Nutrition and Health Post Graduation Program. Nutrition School (FANUT), Federal University of Goias (UFG), Rua 236, nº 343, Setor Universitário, Goiania, GO 74610-070 Brazil; 2 Hypertension League, Federal University of Goias (UFG), Goiania, GO Brazil; 3 Division of Cardiovascular Medicine, Brigham & Women’s Hospital, 75 Francis Street, Boston, MA 02115 USA

**Keywords:** Body mass index, Waist circumference, Weight gain, Obesity

## Abstract

**Background:**

Obesity is a chronic complex disease with an increasing prevalence around the world. Prospective studies in adult cohorts are needed to provide information about predictors of new-onset overweight/obesity on population-based levels. The aim of this study was to identify factors associated with the risk of an adult individual become overweight/obese after 13 years of follow-up.

**Methods:**

Second phase of an observational population-based prospective cohort study in a small town in the Midwest region of Brazil. A representative sample of the adult population (≥18 years) was assessed in 2002 (phase 1). Anthropometric, sociodemographic, dietary intake and lifestyle data were collected. After 13 years of follow-up (2015), the same variables were re-evaluated (phase 2). New-onset overweight/obesity was the outcome variable.

**Results:**

A total of 685 subjects were included with a mean age in phase 1 of 42.7 ± 13.8 years and 56.1 ± 13.8 years in phase 2, the mean follow-up time was 13.2 years and female sex counted for 66.3% of the sample. Total weight gain was 5.9 ± 10.2 Kg, body mass index increased 2.6 ± 3.8 Kg/m^2^ and waist circumference (WC) values increased 8.0 ± 10.5 cm. The prevalence of overweight/obesity went from 49.1% in phase 1 to 69.8% in phase 2 (*p* < 0.001). The factors associated with a decreased risk of new-onset overweight/obesity were ages between 50 and 64 (RR 0.40; CI 0.24–0.67 – *p* = 0.001) and ≥65 years (RR 0.15; CI 0.06–0.35 - *p* < 0.001), being part of the second quartile of fat consumption (RR 0.59; CI 0.35–0.97 – *p* = 0.041), no alcohol consumption (RR 0.59; CI 0.37–0.93 – *p* = 0.024) and smoking (RR 0.58; CI 0.39–0.86 – *p* = 0,007) in phase 1.

**Conclusions:**

We identified in thirteen years of follow-up that older ages, a moderate fat consumption compared to low consumption, no alcohol consumption and smoking habit were related to a decreased risk of new-onset overweight/obesity. Obesity prevention actions must focus on subjects at younger ages and include policies to reduce alcohol consumption.

## Background

Obesity is a chronic complex disease characterized by excessive adipose tissue [1]. The rising prevalence of overweight and obesity in several countries has been described as a global pandemic [2]. Worldwide, the proportion of adults with a body-mass index (BMI) ≥ 25 kg/m2 increased between 1980 and 2013 from 28.8% to 36.9% in men, and from 29.8% to 38.0% in women [2]. In 2014, 52.5% of the Brazilian population was overweight and 17.9% of those were obese [3].

Obesity and overweight are associated with many co-morbidities such as cardiovascular disease, diabetes, hypertension and several of the commonest forms of cancer [4]. Increased weight gains in adult life are related to metabolic syndrome [5], increased risk of stroke [6] and total mortality [7], in late stages of life.

The increasing prevalence of overweight and obesity has been attributed to quantitative and qualitative changes in diet (i.e., higher energy density, more fat, and added sugars in foods, greater saturated fat intake, and reduced intakes of complex carbohydrates, dietary fiber, fruit, and vegetables) in addition to reduced physical activity at work and during leisure time [8]. Ultimately, the main cause leading to excessive weight gain is the imbalance between amount of calories consumed and spent [9].

Sociodemographic characteristics as income, educational level [10], dietary behavior [8, 11–14] and physical activity [15] were previously reported as being associated with weight gain in adult life. Despite that, no national success stories have been reported in the past years on strategies of decreasing population obesity growth [2].

Prospective studies in adult cohorts are needed to provide information about predictors of new onset of overweight/obesity on population-based levels. The necessity of this kind of data is even more meaningful in low to middle income countries, where obesity prevalence growth is still increasing [16].

Considering the exposed, three aspects lead to this study: the need of continuous research on obesity as a public health problem; the lack of longitudinal studies in low and middle income countries related to the subject; and the importance of identifying factors associated to the risk of new cases of overweight/obesity in order to implement effective preventive strategies at a Health system level. So the primary objective of this study was to assess the association of sociodemographic variables, dietary behavior and lifestyle with the risk of new onset of overweight/obesity in adults from a small Brazilian city in the Midwest of the country in a 13 years follow-up period.

The study hypothesis was that age, socioeconomic conditions, food intake patterns and lifestyle habits would be associated with an increased risk of new overweight/obesity cases. These findings would allow health policy strategies against population basis weight gains to be more adequately addressed.

## Methods

The present study is the second phase of an observational population-based prospective cohort study. The first phase in which the cohort was built took place in 2002 in Firminópolis, a small town in the Midwest Region of Brazil. The town had 9666 inhabitants at the period and the study included a representative sample of adult (≥ 18 years) individuals who lived in the urban area of the town. This geographic location was selected because Brazilian epidemiological data on cardiovascular risk at time of phase 1 were missing in some regions of the country [17].

The initial sample size for phase 1, was calculated considering the total city population in 2002, the prevalence of hypertension of 25%, the 95% confidence interval and an estimation error of 10%, which resulted in *n* = 1030. An additional 20% was added to this total to cover eventual losses (*n* = 1236). The final sample consisted of 1167 individuals (430 men and 737 women) [18].

This study was approved by the Ethics in Research Committee from the Federal University of Goias Clinics Hospital (CEP/HC-UFG) with the registration number 396.839. The study followed the humans research regulations according to the National Health Council Resolution number 466/2012. The interviews were conducted after the Consent Form had been signed.

The original sample size was 1167 subjects who were included in the first phase. From those subjects 482 were excluded from phase 2. The reasons for exclusion were: 190 moved to another city, 12 not found at their homes, 10 refused to participate, 100 subjects with no information, 149 excluded due to death, 11 due to physical or mental incapacity and 10 due to incomplete data on weight and height. The final sample size of this study was 685 subjects.

In 2015 subjects were re-assessed on sociodemographic (age, sex, marital status, educational level, income), anthropometric (weight, height, waist circumference), dietary pattern (fat, sugar, fruits and vegetables consumption) and lifestyle (physical activity, alcohol consumption and smoking) variables.

The sociodemographic characteristics analyzed were sex, age (categorized in age groups: 18–33, 34–49, 50–64, ≥65 years), educational level (categorized in years of education: 0, 1–8, 9–11, >11 years), marital status (with or without partner) and income per capita (categorized in quartiles of minimum wage).

The food consumption variables assessed were: habit of removing the meat fat and chicken skin (yes, no and occasionally), habit of eating in front of television (yes, no, occasionally), number of daily meals (total number, categorized in 1–4 and ≥4 meals), fruits, vegetables, sugar and fat consumption (consumption score categorized by quartiles). The food consumption evaluation was performed with a frequency of food intake questionnaire, in which eight categories of consumption were used for classification (never, less than once a months, 1 to 3 times a month, once a week, 2 to 3 times a week, 4 to 6 times a week, once a day, 2 or more times a day). The frequency of food consumption was converted into scores, as proposed by Formes [19] et al. This score represented the mean daily consumption frequency of the food groups.

The foods evaluated in each food group were: fruits (avocado, acai, pineapples, melon, water melon, banana, orange, tangerine, apple, pearl, papaya, mango, guava, grapes, kaki, peach and strawberry), vegetables (lettuce, cabbage, chard, watercress, arugula, kale, spinach, mustard, chicory, tomato, cucumber, broccoli, zucchini, scarlet eggplant, pumpkin, beet e carrot), sugars (chocolate, candy, cake, donut, ice cream, pop stickle, sugar, honey, guava sweet, pudding, sugar cane juice sweet and milk jam) and fats (sour cream, butter, regular margarine, light margarine, mayonnaise, oil, olive oil, pork fat and bacon).

Variables concerning life habits were: smoking (current smoker; non-smoker or ex-smoker); alcohol consumption (consumption or no consumption of alcoholic beverages, regardless of the frequency and amount), watching television habit (categorized in ≤2 h/day and >2 h/day) and physical activity (at leisure time, commuting and at work).

Physical activity at leisure time was classified as sedentary, mild, moderate or vigorous. Commuting physical activity was classified in less than 15 min and 15 min or more of exercise to get to work. Physical activity at work was classified as: sedentary, mild, moderate and vigorous.

Weight was measured with individuals in orthostatic position, with arms extended along body, barefoot and wearing light clothes. A PLENA scale, model GIANT LITHIUM, with a maximum capacity of 150 kg and a precision of 100 g was used to measure weight. A SECCA stadiometer, model 206, with a precision of 0.1 cm was used to measure height, with participants standing barefoot.

Body mass index (BMI) was calculated using weight in kilograms divided by height in square meters (kg/m^2^). The BMI values were classified as BMI < 24.9 kgm^2^ (low weight/normal weight); BMI from 25 to 29.9 (overweight) and BMI > 30 kgm^2^ (obesity) [20].

Waist circumference (WC) was measured using an inextensible measuring tape, with patient in standing position, with arms extended along the body, wearing as little clothing as possible and measured in the horizontal plane in the midpoint between the lateral iliac crest and the last rib. It was classified as normal, increased and substantially increased according to the values <94 cm, between 94 and 102 cm and >102 cm for men; < 80, between 80 and 88 cm and >88 cm for women [21].

The anthropometric variables body weight, BMI and WC were handled as continuous and as categorical variables. Weight gain, increase of BMI and WC were obtained from the differences between the measurements in phase 2 and phase 1. The outcome “overweight/obesity” was dichotomized in “incidence” and “non-incidence”. The incidence of overweight/obesity was considered for those individuals who switched from the normal weight category to overweight or obesity and those who went from overweight to obesity.

Statistical analyses were performed with the software SPSS version 21. Normality was tested with Kolmogorov-Smirnov test. Paired T-test (continuous variables) and McNemar test (categorical variables) were used to verify differences between the two moments of data collection. Bivariate and multivariate logistic regressions were used to verify which variables from phase 1 were predictors of overweight/obesity incidence. Variables with a *p*-value <0.20 in the bivariate regression were included in the multivariate regression, by the stepwise backward method (Wald test). The final regression model was adjusted for the variables sex, follow-up time, initial BMI and WC. Significance level was set as 5% and 95% confidence interval.

## Results

The mean age in phase 1 was 42.7 ± 13.8 years while in in phase 2 it went to 56.1 ± 13.8 years. The mean follow-up time was 13.2 years and female sex represented 66.3% (*n* = 454) of the sample. Total weight gain was 5.9 ± 10.2 Kg, mean BMI increased 2.6 ± 3.8 Kg/m^2^ and mean waist circumference values increased 8.0 ± 10.5 cm. A daily energy expenditure decrease was observed in this sample, since sedentary lifestyle in work increased, the proportion of subjects who spent more than 15 min commuting decreased, the number of subjects watching ≥2 h per day of television increased and no changes in the pattern of leisure physical activity was observed. The overall study population characteristics in the two assessments phases are listed in Table 1.

**Table 1:** Sociodemographic, anthropometric, food consumption and lifestyle variables in Phase 1 and Phase 2. Firminopolis, Brazil (2002–2015)

Variables	*Number*	Phase 1	Phase 2	*p*
Age, years (mean ± SD)	685	42.7 ± 13.8	56.1 ± 13.8	<0.001*
Years of education (mean ± SD)	685	6.0 ± 4.1	6.8 ± 4.5	<0.001*
Per capita income, MW (mean ± SD)	685	1.0 ± 1.1	1.2 ± 0.9	<0.001*
Marital status, n (%)	678			
With partner		486 (71.7%)	456 (67.3%)	<0.001*
Without partner		192 (28.3%)	222 (32.7%)	<0.001*
Weight, kg (mean ± SD)	685	64.7 ± 13.3	70.6 ± 15.6	<0.001*
Body mass index, kg/m^2^ (mean ± SD)	685	25.3 ± 4.6	27.9 ± 5.3	<0.001*
Waist Circumference, cm (mean ± SD)	685	85.2 ± 11.3	93.2 ± 12.6	<0.001*
Daily meals (mean ± SD)	685	3.6 ± 0.9	4.1 ± 1.0	<0.001*
Eats watching TV, n (%)	674			
Yes		181 (26.9%)	162 (24.0%)	0.169
No		318 (47.2%)	364 (54.0%)	<0.001*
Occasionally		175 (26.0%)	148 (22.0%)	0.048*
Remove meat fat, n (%)	678			
Yes		302 (44.5%)	352 (51.9%)	<0.001*
No		301 (44.4%)	234 (34.5%)	<0.001*
Occasionally		75 (11.1%)	92 (13.6%)	0.134
Remove chicken skin, n (%)	677			
Yes		338 (49.9%)	379 (56.0%)	<0.001*
No		290 (42.8%)	248 (36.6%)	0.002*
Occasionally		49 (7.2%)	50 (7.4%)	0.914
Daily fruits consumption (mean ± SD)	685	1.0 ± 0.8	1.1 ± 0.8	<0.001*
Daily vegetables consumption (mean ± SD)	685	2.2 ± 1.4	2.2 ± 1.4	0.668
Daily sugar consumption (mean ± SD)	685	1.7 ± 0.8	1.3 ± 0.8	<0.001*
Daily fat consumption (mean ± SD)	685	2.1 ± 0.8	2.5 ± 0.9	<0.001*
Commuting PA^a^, n (%)	684			
< 15 min		529 (77.3%)	581 (84.9%)	<0.001*
> 15 min		155 (22.7%)	103 (15.1%)	<0.001*
Occupational PA^a^, n (%)	685			
Sedentary		457 (66.7%)	528 (77.1%)	<0.001*
Mild		142 (20.7%)	84 (12.3%)	<0.001*
Moderate		49 (7.2%)	33 (4.8%)	0.069
Vigorous		37 (5.4%)	40 (5.8%)	0.727
Leisure-time PA^a^, n (%)	685			
Sedentary		437 (63.8%)	446 (65.1%)	0.394
Mild		212 (30.9%)	211 (30.8%)	0.944
Moderate		36 (5.3%)	28 (4.1%)	0.297
Hours watching TV/day, n (%)	680			
≤ 2		489 (71.9%)	368 (54.1%)	<0.001*
> 2		191 (28.1%)	312 (45.9%)	<0.001*
Alcohol consumption, n (%)	685			
Yes		219 (32.0%)	173 (25.3%)	0.002*
No		466 (68.0%)	512 (74.7%)	<0.001*
Smoking, n (%)	685			
Non smoker		391 (57.1%)	386 (56.4%)	0.678
Current		146 (21.3%	84 (12.3%)	<0.001*
Ex smoker		148 (21.6%)	215 (31.4%)	<0.001*

^a^*PA* physical activity, *Statistically significant at α = 0.05

**Fig. 1:**
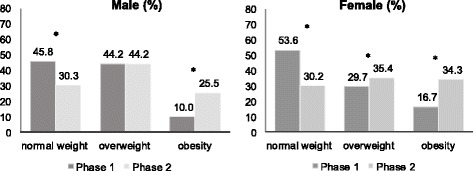
Nutritional status evolution based on body mass index (*n* = 685). Firminopolis, Brazil (2002–2015). *Statistically significant at α = 0.05

In the overall sample the rate of normal weight went from 50.9% to 30.2% (*p* < 0.001), overweight from 34.6% to 38.4% (*p* = 0.067) and obesity increased from 14.5% to 31.4% (*p* < 0.001). When the sexes were assessed separately the rates of normal weighted individuals decreased while obesity increased in the thirteen years interval between the two evaluations. For overweight, its prevalence kept the same in men and increased in women as shown in Fig. 1.

**Fig. 2:**
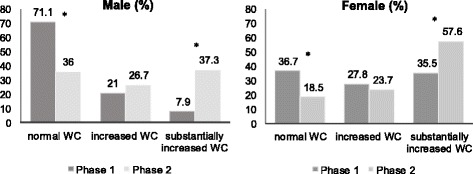
Nutritional status evolution based on waist circumference (*n* = 685). Firminopolis, Brazil (2002–2015). *Statistically significant at α = 0.05

Considering WC the rates of subjects with normal WC went from 48.2% to 24.4% (*p* < 0.001), increased WC from 25.6% to 24.8% (*p* = 0.718) and the substantially increased WC from 26.2% to 50.8% (*p* < 0.001). The same pattern of decreasing rates of normal WC, non-significant changes in increased WC and growing rates of substantially increased WC were found in males and females when analyzed apart. (Fig. 2).

In order to assess the nutritional status evolution according to age category in phase 1, we compared the BMI and WC categories distribution in phase 1 to the distribution in phase 2. The comparison was made between the same subjects and the age category from phase 1 (age in 2002) was used as the reference. The results are presented in Table 2 and Table 3. A decrease in the proportion of subjects in the normal weight and WC categories as opposed to an increase in obesity and substantially increased WC was found in the individuals from the three initial age categories (18–33, 34–49 and 50–64). The only exception to this pattern in these three initial age categories was seen in the obesity prevalence in the 50–64 category which did not change. Oppositely, no changes happened at all in the obesity or substantially increased WC proportion between phase 1 and 2 in subjects ≥65 years. (Table 2 and Table 3).

**Table 2:** Body mass index evolution according to age category in 2002 (*n* = 685). Firminopolis, Brazil (2002–2015)

	Normal Weight			Overweight			Obesity		
Age in 2002 (years)	Phase 1	Phase 2		Phase 1	Phase 2		Phase 1	Phase 2	
	n (%)		*p*	n (%)		*p*	n (%)		*p*
18–33	130 (61.3)	62 (29.2)	<0.001*	68 (32.1)	81 (38.2)	0.18	14 (6.6)	69 (32.6)	<0.001*
34-49	118 (46.8)	65 (25.8)	<0.001*	93(36.9)	99 (39.3)	0.58	41 (16.3)	88 (34.9)	<0.001*
50-64	74 (44.0)	54 (32.1)	0.020*	58 (34.5)	66 (39.3)	0.37	36 (21.4)	48 (28.6)	0.130
>65	27 (50.9)	26 (49.1)	0.850	18 (34.0)	17(32.1)	0.84	8 (15.1)	10 (18.9)	0.110

*Statistically significant at α = 0.05

**Table 3:** Waist circumference evolution according to age category in 2002 (*n* = 685). Firminopolis, Brazil (2002–2015)

	Normal WC^a^			Increased WC^a^			Substantially increased WC^a^		
Age in 2002 (years)	Phase 1	Phase 2		Phase 1	Phase 2		Phase 1	Phase 2	
	n (%)		*p*	n (%)		*p*	n (%)		*p*
18–33	131 (61.8)	62 (29.4)	0.030*	49 (23.1)	60 (28.4)	0.210	32 (15.1)	89 (42.2)	<0.001*
34–49	119 (47.2)	52 (20.9)	<0.001*	69 (27.4)	59 (23.7)	0.350	64 (25.4)	138 (55.4)	<0.001*
50–64	61 (36.3)	34 (20.2)	<0.001*	46 (27.4)	41 (24.4)	0.540	61(36.3)	93 (55.4)	<0.001*
>65	20 (37.7)	18 (34.0)	0.690	10 (18.9)	9 (17.0)	0.810	23 (43.4)	26 (49.1)	0.570

^a^*WC* waist circumference

*Statistically significant at α = 0.05

From 133 new obesity cases diagnosed 31 (23.3%) had normal weight in phase 1 and became obese while 102 (76.7%) had overweight in phase 1 and became obese. Regarding the incidence of substantially increased WC, 83 (43.7%) subjects with normal WC went to substantially increased WC and 107 (56.3%) who had increased WC went to the substantially increased category. The incidence of overweight, obesity, increased and substantially increased WC are presented in Table 4.

**Table 4:** Incidence of overweight, obesity, increased and substantially increased waist circumference in a thirteen years interval (*n* = 685). Firminopolis, Brazil (2002–2015)

Event	New cases	Incidence
	(Number)	(Percent)
Overweight	135	30.1
Obesity	133	22.7
Increased WC^a^	105	20.5
Substantially increased WC^a^	190	37.8

^a^*WC* waist circumference

In the bivariate regression, the variables that showed no association with the outcome were sex (*p* = 0.694), total number of meals a day (*p* = 0.353), meals watching television (*p* = 0.843), habit of removing chicken skin (*p* = 0.285), sugar consumption (*p* = 0.450), vegetables consumption (*p* = 0.850), physical activity while going to work (*p* = 0.541) and leisure physical activity (*p* = 0.421).

In the final adjusted model, among sociodemographic variables, age was the only one statically significant, showing that older individuals in phase 1 (age over 50 years) had less risk of becoming overweight during follow-up. Regarding fat consumption the risk of becoming overweight was lower in subjects from the second quartile when compared to the first quartile. In relation to lifestyle variables, overweight risk was lower in the subjects who did not consume alcohol (*p* = 0.007) and in those who were smokers (*p* = 0.024) in phase 1 (Table 5).

**Table 5:** Relative risk of new-onset overweight/obesity in a thirteen years interval (*n* = 685). Firminopolis, Brazil (2002–2015)

	Bivariate			Multivariable		
	*RR*	*CI*	p*	*RR*	*CI*	p**
Age						
18–33	1.00			1.00		
34–49	0.61	0.41–0.87	0.008	0.77	0.50–1.16	0.217
50–64	0.31	0.20–0.48	<0.001	0.40	0.24–0.67	0.001
≥ 65	0.16	0.07–0.34	<0.001	0.15	0.06–0.35	<0.001
Years of education						
0	1.00			1.00		
1–8	1.67	0.91–3.05	0.097	1.12	0.54–2.30	0.755
9–11	2.22	1.17–4.22	0.014	1.03	0.45–2.36	0.942
≥ 12	2.50	0.89–7.00	0.081	1.39	0.39–4.93	0.603
Marital status						
With partner	1.00			1.00		
Without partner	0.73	0.51–1.03	0.080	0.75	0.50–1.12	0.164
Per capita income (quartiles/MW)						
Q1 (0 ⊢0,5)	1.00			1.00		
Q2 (0,5 ⊢ 0,8)	0.91	0.58–1.40	0.657	0.90	0.54–1.50	0.689
Q3 (0,8 ⊢ 1,1)	0.71	0.46–1.07	0.109	0.83	0.50–1.35	0.461
Q4 (1,1 ⊢ 13,9)	0.85	0.56–1.29	0.459	1.10	0.67–1.83	0.689
Remove fat meat						
Yes	1.00			1.00		
No/Occasionally	1.22	0.89–1.66	0.190	1.10	0.75–1.59	0.620
Fat consumption (quartiles)						
Q1 (0,2 ⊢ 1,4)	1.00			1.00		
Q2 (1,4 ⊢ 2,1)	0.69	0.45–1.06	0.097	0.59	0.35–0.97	0.041
Q3 (2,1 ⊢ 2,5)	0.84	0.54–1.29	0.430	0.78	0.47–1.30	0.350
Q4 (2,5 ⊢ 5,2)	0.77	0.53–1.28	0.403	0.66	0.39–1.09	0.106
Fruit consumption (quartiles)						
Q1 (0,2 ⊢ 0,4)	1.00			1.00		
Q2 (0,4 ⊢ 0,8)	1.35	0.87–2.10	0.178	1.32	0.79–2.20	0.279
Q3 (0,8 ⊢ 1,4)	1.36	0.88–2.11	0.164	1.38	0.83–2.32	0.210
Q4 (1,4 ⊢ 5)	1.18	0.76–1.84	0.453	1.18	0.69–2.00	0.531
Occupational PA						
Sedentary	1.00			1.00		
Mild	1.46	1.00–2.14	0.049	1.29	0.80–2.07	0.294
Moderate/intense	1.25	0.78–1.99	0.350	1.18	0.64–2.16	0.582
Television hours/day						
≤ 2	1.00			1.00		
> 2	1.26	0.90–1.77	0.173	0.99	0.66–1.48	0.989
Alcohol consumption						
Yes	1.00			1.00		
No	0.58	0.42–0.80	0.001	0.58	0.39–0.86	0.007
Smoking						
Never	1.00			1.00		
Smoker	0.78	0.52–1.15	0.190	0.59	0.37–0.93	0.024
Ex-smoker	0.60	0.40–0.89	0.012	0.82	0.50–1.33	0.425

*RR* Relative Risk, *CI* Confidence Interval, *MW* minimum wage, *PA* physical activity

*p value <0.20 for the variables tested in the bivariate regression

**p value of the variables added to the multivariate regression model, adjusted for sex, follow-up time, initial BMI and initial WC

## Discussion

In this second phase of an observational population-based prospective cohort study in a small town from a middle income country with a mean follow-up of 13 years we assessed the risk predictors of becoming overweight/obese. No factors were independently associated with getting overweight/obese in a thirteen years follow-up period, but older ages, a low/moderate fat consumption, no alcohol consumption and current smoking habit were related to a decreased risk of becoming overweight/obese along this period.

The prevalence of overweight/obesity in adults from Firminopolis in 2015 was 69.8% (overweight =38.4% and obesity = 31.4%). These rates are considered high when compared to national [19] and international data [22]. The mean weight gain was 5.95 kg in 13 years (5.52 kg in men and 6.17 kg in women). If we divide this value for 13 years, the mean weight gain per year was 457 g. Variable amounts of weight gain per year have been reported previously, depending on the population included in the sample selection [14, 23].

Age was a variable related to the risk of overweight/obesity. This risk decreased with ageing, considering the age groups from 50 to 64 and ≥65 years. This pattern had already been reported in the literature [24] and may be related to the weight loss observed in elderly subjects. Weight gain associated with ageing usually happens until 55–60 years. After that, the body composition changes with a decrease in lean body mass and an increase in body fat, leading to weight loss. This pattern is more evident in women [25]. This weight loss can also be explained by chronic diseases, aspects related to socioeconomic and familial conditions, partner loss, depression, social isolation, lack of social integration, as well as changes in chewing and in the sensorial perception of food [26].

Subjects in the second quartile of fat consumption had lower risk of developing overweight/obesity, when compared to those at the first quartile. The excessive consumption of fat is associated to weight gain, due to the high energetic density of fatty foods [22]. Nevertheless it is important to notice that subjects at the second quartile of fat consumption had the habit of eating fat 1.4 to 2.1 times a day. If the subject had lunch and dinner at home, he would be included at the second quartile, as he would use some source of fat for cooking those meals. By saying that, it is not accurate to state that fat consumption of the second quartile was high, and it even follows the recommendations of fractioning the meals [27]. Either way the public health recommendations should consider the reduction of fatty foods consumption in order to maintain adequate body weight, particularly for the sedentary subjects and those with a genetic disposition to obesity [12].

Subjects who did not consume alcohol on phase 1 had 42% less risk of becoming overweight/obese when compared to those who did consume alcohol. Prospective studies assessing the alcohol consumption effect over the outcome overweight/obesity are scarce. The results of the present study are concordant with a cross-sectional study that reported positive association between alcohol consumption and abdominal as well as general adiposity [28]. It has been suggested that adiposity deposits in subjects who consume alcohol occur preferentially at the abdominal region [29]. Alcohol may change lipids oxidation, since it has metabolism priority, contributing to fat stocking, particularly in the abdominal area. This fact can explain the significant association between excessive alcohol consumption and the abdominal fat variables [30]. Oppositely a study showed that high alcohol consumption was inversely associated to abdominal adiposity gain, and the authors’ attributed their results to the thermogenic effect of the enzyme alcohol-dehydrogenase [23].

Alcohol consumption by itself, favors weight gain since it adds additional calories to daily individual calories consumption. It is ranked in second place in the energetic density hierarchy (7 kcal/g) [31], and besides that, alcohol consumption is associated to concomitant ingestion of other foods.

Regarding smoking, individuals in phase 1 who smoked had 41% less risk of becoming overweight/obese during the follow-up period when compared to those who never smoked. Although this result is concordant to previously published studies [32, 33], this association is still conflicting. Opposite results were also published before, with a higher weight gain in smokers when compared to non-smokers [34].

There is evidence in the literature that subjects who quit smoking have a higher chance to weight gain when compared to current smokers and those who start smoking [13]. Nevertheless in our study the ex-smokers from phase 1 did not have a higher risk of becoming overweight/obese throughout the years.

Nicotine effects over the body are the most important factors why smokers have less risk of weight gain than non-smokers. Nicotine acts as an appetite suppressor, generates satiety and gastric fullness sensations, inhibits food consumption [35] and increases metabolic basal rates [23]. Despite these effects, it is important to continuously recommend smoking cessation, considering the harmful body effects of nicotine and other toxic components of cigarettes [36]. It is worth highlighting that from 2002 to 2015/16 there was a smoking rate decrease in Firminópolis.

An important point to be mentioned is that subjects with BMI < 18.5 kg/m^2^ (low weight) were included in the normal weight category whereas the main goal of the study was overweight/obesity incidence. Another reasonable explanation to this inclusion is the small number of subjects with low weight when compared to the other categories. In 2002 from the 685 subjects, 31 had BMI < 18.5 kg/m^2^ and in 2015 only 12.

The scarcity of longitudinal studies focusing on overweight/obesity incidence compromised the comparison with similar data and was a difficulty the authors of this study faced. Although the impossibility of finding all individuals from the initial sample was a limitation, it was minimized by the baseline characteristics comparison of individuals not found with those with complete follow-up. By comparing the subjects assessed in phase 2 (*n* = 685) and the non-assessed (losses and exclusions = 482) no differences were observed between groups characteristics (*p* > 0.05). Non-paired T-test and Chi-Square test were used for this analysis.

Facing such a complex problem as overweight/obesity and finding conflicting results in the literature leads to the conclusion that more longitudinal studies are needed addressing weight gain and its predictors. Cohort studies with multiple evaluations throughout the years are excellent options and need to be encouraged. These contemporary cohorts, exposed to the nutritional reality from westernized countries (abundant energy-rich processed foods availability) [37] will help the healthcare community to better understand the causes of obesity, leading to a more effective management and control of the condition.

It is clear, on the other hand, that public health actions to prevent obesity must be implemented focusing on individuals at younger ages, as well as including alcohol consumption as part of the problem. This approach will probably be more effective in educating the population to adopt health behaviors that in the long term will change the incidence of overweight/obesity and avoid it epidemic condition as seen nowadays.

## Conclusion

In conclusion we did not identify any risk factor independently associated with getting overweight/obese in thirteen years of follow-up, but older ages, a low/moderate fat consumption, no alcohol consumption and current smoking habit were related to a decreased risk of becoming overweight/obese along this period.

## Abbreviations

BMI: Body mass index
CI: Confidence interval
MW: Minimum wage
PA: Physical activity
RR: Relative risk
SD: Standard deviation
SPSS: Statistical Package for the Social Sciences
WC: Waist circumference
